# Regeneration of *Phaseolus vulgaris* from epicotyls and hypocotyls *via* direct organogenesis

**DOI:** 10.1038/s41598-019-42723-8

**Published:** 2019-04-18

**Authors:** Katarzyna Hnatuszko-Konka, Tomasz Kowalczyk, Aneta Gerszberg, Sława Glińska, Izabela Grzegorczyk-Karolak

**Affiliations:** 1 Department of Molecular Biotechnology and Genetics, Faculty of Biology and Environmental Protection, University of Lodz, 90-237 Lodz, Banacha 12/16, Poland; 2 Laboratory of Microscopic Imaging and Specialized Biological Techniques, Faculty of Biology and Environmental Protection, University of Lodz, 90-237 Lodz, Banacha 12/16, Poland; 3 Department of Biology and Pharmaceutical Botany, Medical University of Lodz, 90-151 Lodz, Muszynskiego 1, Poland

**Keywords:** Plant biotechnology, Plant regeneration

## Abstract

The tissue culture of *Phaseolus vulgaris* has always been considered difficult. Its regenerative capacity and response to culture conditions are highly genotype-dependent and hamper the application of genetic engineering. The objective of this study was to develop a repeatable technique for organogenic bud induction from selected explants of the common bean. Epicotyls and hypocotyls of six cultivars were investigated to determine the effect of the genotype, and four variants of two basal media (Murashige-Skoog and Gamborg) were tested. The composition of these medium variants was based on the published data suggesting the most universal medium compounds that show the advantage of being applicable to different cultivars. As a result, the common bean epicotyls showed undisputed regeneration superiority over the hypocotyls. Moreover, a well-known observation was confirmed, namely that common bean regeneration is cultivar-specific or at least specific to the cluster of related cultivars. However, efficient regeneration was achieved most often when the epicotyls were incubated on the MS or B5 media amended with AgNO_3_ and BAP. Additionally, the positive synergistic influence of activated charcoal and silver nitrate on bud formation was demonstrated. The highest values of the epicotyl *in vitro* response for the common bean cultivars could be presented as follows: Czerwona (70.00%) > Goldpantera (58.89%) and Ibiza (58.89%) > Plus (55.56%) > Laponia (50.56%) > Złota Saxa (46.11%).

## Introduction

The common bean is the most widely cultivated of *Phaseolus* species worldwide, since it is a very important source of protein and calories. This warm-season legume also plays an important role as a natural system of nitrogen-fixation. However, a number of biotic and abiotic stresses severely affect the yield of this crop. *In vitro* regeneration of the common bean is frequently considered to be difficult, which significantly hampers progress in the transformation. *Phaseolus vulgaris* displays extremely high diversity regarding the response to tissue culture conditions, hence a cultivar/genotype-specific protocol of regeneration is suggested^1^. It was demonstrated, for example, in the contrasting results reported by Delgado-Sanchez *et al*.^2^ and Gatica Arias *et al*.^3^ regarding adenine usage. Of course, this genotype-dependent response is also determined by the type or state of the explant, cell or tissue specialization and by the cultivation conditions^4^. Nevertheless, the common bean was regenerated *via* organogenic^5–7^ and embryogenic pathways^8–10^ from different plant explants: intact seedlings, embryonic axes, cotyledons, cotyledonary nodes, leaf petioles, internodes, auxiliary shoots, hypocotyls or leaves^3,11–13^. However, plant regeneration employing the published protocols remains difficult to reproduce.

The objective of the reported study was to develop a repeatable technique for organogenic bud induction from selected explants of young and mature plants of the common bean. However, the assumption was made that different published protocols could be retested to optimize them and check their usefulness regarding chosen cultivars and explants. The plant material included six varieties: Czerwona, Plus, Laponia, Złota Saxa, Ibiza and Goldpantera (the last four are listed in *The Common Catalogue of Varieties of Vegetable Species*, 35th complete edition, 2016)^14^. The studies were conducted on randomly chosen cultivars as a starting point, following Malmberg’s^15^ suggestion, assuming that the wide screening of genetic lines might become a base of knowledge for plant regeneration^1,4^. It should be noted here that different parts of young and mature plants were tested in our research prephase (preliminary investigation of the regeneration capacity tested on a small plant pool, 20 explants, *data not shown*). Finally, explants such as leaves, roots or stems of mature plants were excluded from the final experiment, as were cotyledons and the roots of seedlings. Therefore, we report an investigation of the regeneration potential of seedling epicotyls and hypocotyls from different cultivars of *Phaseolus vulgaris*.

The final *formulae* tested were planned to be the result of the chosen plant explant, successful methods available in the literature and the specific response of the particular variety obtained in the preliminary phase of research. By that means, finally four variants for two basal media (full Murashige-Skoog – MS and Gamborg – B5 media) were proposed and tested (Table 1). The use of both was motivated by the considerably large differences in their regeneration triggering efficiency^6^. The compositions of our variants were based on the published data suggesting the most universal medium compounds that show the advantage of being applicable to different cultivars (although we knew that in some cases different explants were used). The first variant of both basal media is free of phytohormons or ethylene inhibitors. The next three are supplemented with phytoregulators, according to the protocols produced by Ahmed *et al*.^16^, Delgado-Sanchez *et al*.^2^ and to a certain extent by Kwapata *et al*.^8^, respectively. Those reports constituted the starting points in our research prephase. Ahmed *et al*.^16^ recommended tissue culture on the MS medium combined with 1 mg/L BAP and 0.1 mg/L NAA for multiple shoot induction from cotyledonary nodes and we adopted his medium composition directly as variant II. We applied one of the medium variants investigated by Delgado-Sanchez *et al*.^2^ in a very similar way. The authors observed the formation of organogenic buds of the common bean only when BAP concentrations of 5 and 10 mg/L were applied. Treatments with a low concentration (0.0; 0.1 mg/L) induced only root and stem elongation. On the contrary, the application of adenine at none of the concentrations tested produced any significant increase in bud formation. Since that was also our observation in all the cultivars used during the research prephase (*data not shown*), the recommended BAP concentration (10 mg/L) without the adenine hemisulfate component was established as variant III. The last variant *formula* was inspired by our preliminary research and the paper by Kwapata *et al*.^8^, who regenerated multiple shoots on the MS medium containing 2.5 mg/L BA and 0.1 mg/L IAA supplemented with absorbent (15 mg/L activated charcoal) or antioxidant (30 mg/L silver nitrate) to inhibit the effect of phenolic compounds. Consequently, as we did not observe a significant effect of IAA presence, variant IV was established as a combination of compounds presented in Table 1. Kwapata *et al*.^8^ proposed a useful method to limit phenolic compounds, which in our experiment had a beneficial influence on explant condition at the level of preliminary testing of the media. Therefore, we decided to supply all the variants of the media with activated charcoal in an optimized concentration.

**Table 1:** The tested variants of the induction–multiplication medium (Murashige-Skoog I–IV and Gamborg Medium I–IV).

I	MS + 3% sucrose + 0.4% activated charcoal	starting point
II	MS + 3% sucrose + 0.4% activated charcoal + 0.1 mg/L NAA + 1 mg/L BAP	based on Ahmed *et al*. 2002
III	MS + 3% sucrose + 0.4% activated charcoal + 10 mg/L BAP	based on Delgado-Sánchez *et al*., 2006
IV	MS + 3% sucrose + 0.4% activated charcoal + 10 µM AgNO_3_ + 2.5 mg/L BAP	based on Kwapata *et al*. 2010
I	B5 + 3% sucrose + 0.4% activated charcoal	starting point
II	B5 + 3% sucrose + 0.4% activated charcoal + 0.1 mg/L NAA + 1 mg/L BAP	based on Ahmed *et al*. 2002
III	B5 + 3% sucrose + 0.4% activated charcoal + 10 mg/L BAP	based on Delgado-Sánchez *et al*., 2006
IV	B5 + 3% sucrose + 0.4% activated charcoal + 10 µM AgNO_3_ + 2.5 mg/L BAP	based on Kwapata *et al*. 2010

## Results

**Figure 1:**
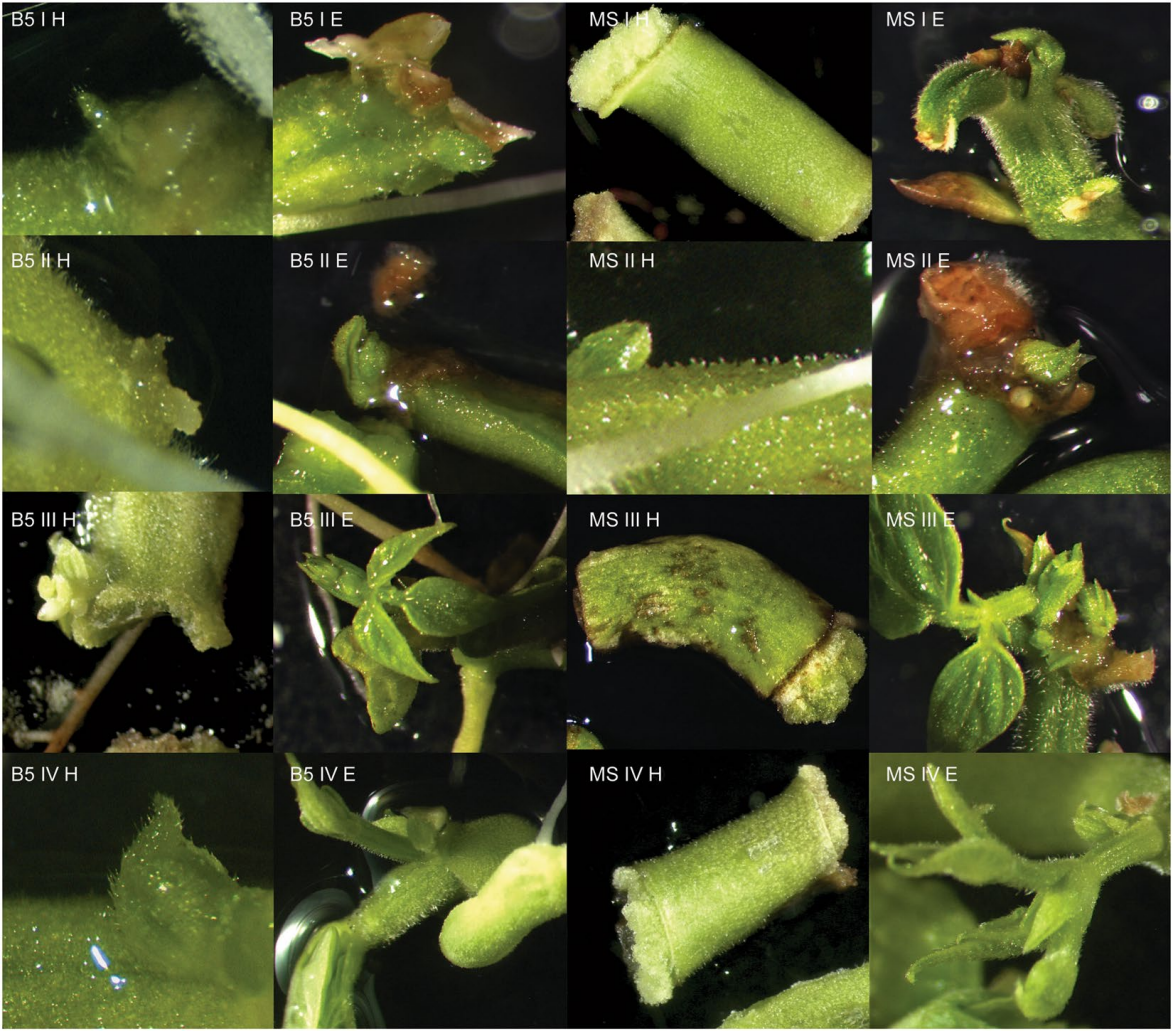
*In vitro* response of the hypocotyls and epicotyls of the Goldpantera cultivar on different media. B5 I H – Gamborg Medium, variant I, hypocotyl; B5 II H – Gamborg Medium, variant II, hypocotyl; B5 III H – Gamborg Medium, variant III, hypocotyl; B5 IV H – Gamborg Medium, variant IV, hypocotyl; B5 I E – Gamborg Medium, variant I, epicotyl; B5 II E – Gamborg Medium, variant II, epicotyl; B5 III E – Gamborg Medium, variant III, epicotyl; B5 IV E – Gamborg Medium, variant IV, epicotyl; MS I H – Murashige-Skoog Medium, variant I, hypocotyl; MS II H – Murashige-Skoog Medium, variant II, hypocotyl; MS III H – Murashige-Skoog Medium, variant III, hypocotyl; MS IV H – Murashige-Skoog Medium, variant IV, hypocotyl; MS I E – Murashige-Skoog Medium, variant I, epicotyl; MS II E – Murashige-Skoog Medium, variant II, epicotyl; MS III E – Murashige-Skoog Medium, variant III, epicotyl; MS IV E – Murashige-Skoog Medium, variant IV, epicotyl.

**Figure 2:**
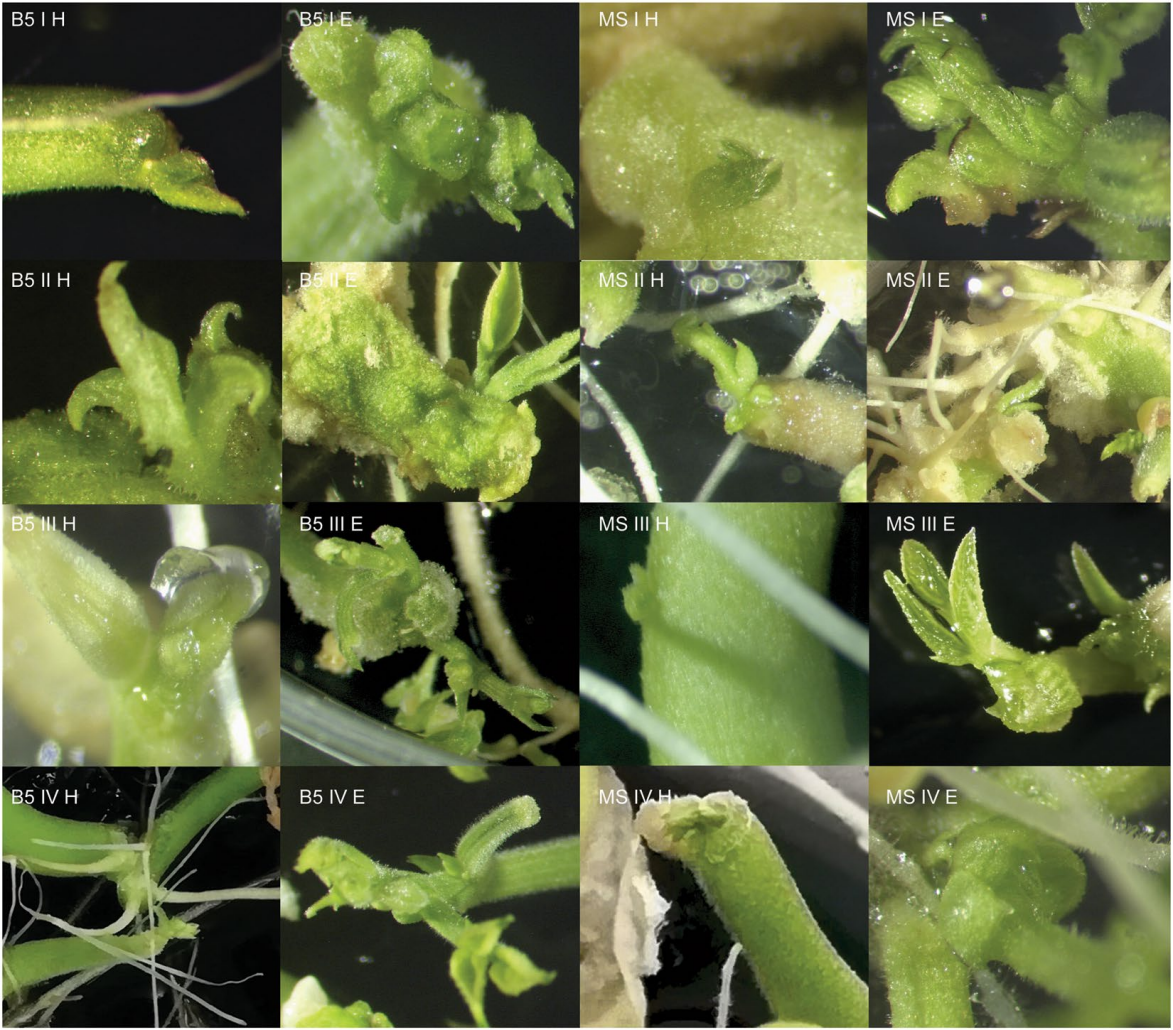
*In vitro* response of the hypocotyls and epicotyls of the Złota Saxa cultivar on different media. B5 I H – Gamborg Medium, variant I, hypocotyl; B5 II H – Gamborg Medium, variant II, hypocotyl; B5 III H – Gamborg Medium, variant III, hypocotyl; B5 IV H – Gamborg Medium, variant IV, hypocotyl; B5 I E – Gamborg Medium, variant I, epicotyl; B5 II E – Gamborg Medium, variant II, epicotyl; B5 III E – Gamborg Medium, variant III, epicotyl; B5 IV E – Gamborg Medium, variant IV, epicotyl; MS I H – Murashige-Skoog Medium, variant I, hypocotyl; MS II H – Murashige-Skoog Medium, variant II, hypocotyl; MS III H – Murashige-Skoog Medium, variant III, hypocotyl; MS IV H – Murashige-Skoog Medium, variant IV, hypocotyl; MS I E – Murashige-Skoog Medium, variant I, epicotyl; MS II E – Murashige-Skoog Medium, variant II, epicotyl; MS III E – Murashige-Skoog Medium, variant III, epicotyl; MS IV E – Murashige-Skoog Medium, variant IV, epicotyl.

**Figure 3:**
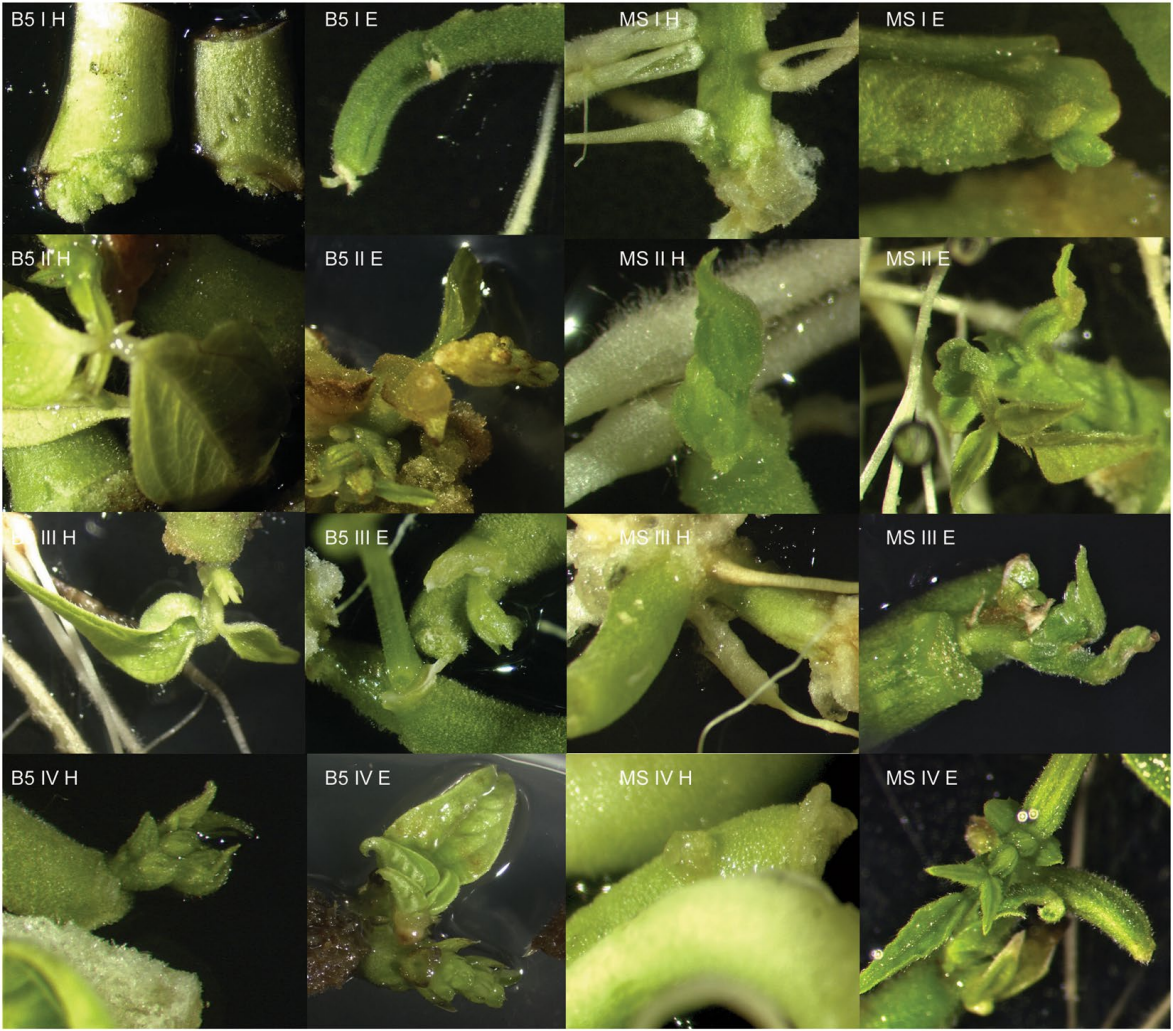
*In vitro* response of the hypocotyls and epicotyls of the Laponia cultivar on different media. B5 I H – Gamborg Medium, variant I, hypocotyl; B5 II H – Gamborg Medium, variant II, hypocotyl; B5 III H – Gamborg Medium, variant III, hypocotyl; B5 IV H – Gamborg Medium, variant IV, hypocotyl; B5 I E – Gamborg Medium, variant I, epicotyl; B5 II E – Gamborg Medium, variant II, epicotyl; B5 III E – Gamborg Medium, variant III, epicotyl; B5 IV E – Gamborg Medium, variant IV, epicotyl; MS I H – Murashige-Skoog Medium, variant I, hypocotyl; MS II H – Murashige-Skoog Medium, variant II, hypocotyl; MS III H – Murashige-Skoog Medium, variant III, hypocotyl; MS IV H – Murashige-Skoog Medium, variant IV, hypocotyl; MS I E – Murashige-Skoog Medium, variant I, epicotyl; MS II E – Murashige-Skoog Medium, variant II, epicotyl; MS III E – Murashige-Skoog Medium, variant III, epicotyl; MS IV E – Murashige-Skoog Medium, variant IV, epicotyl.

**Figure 4:**
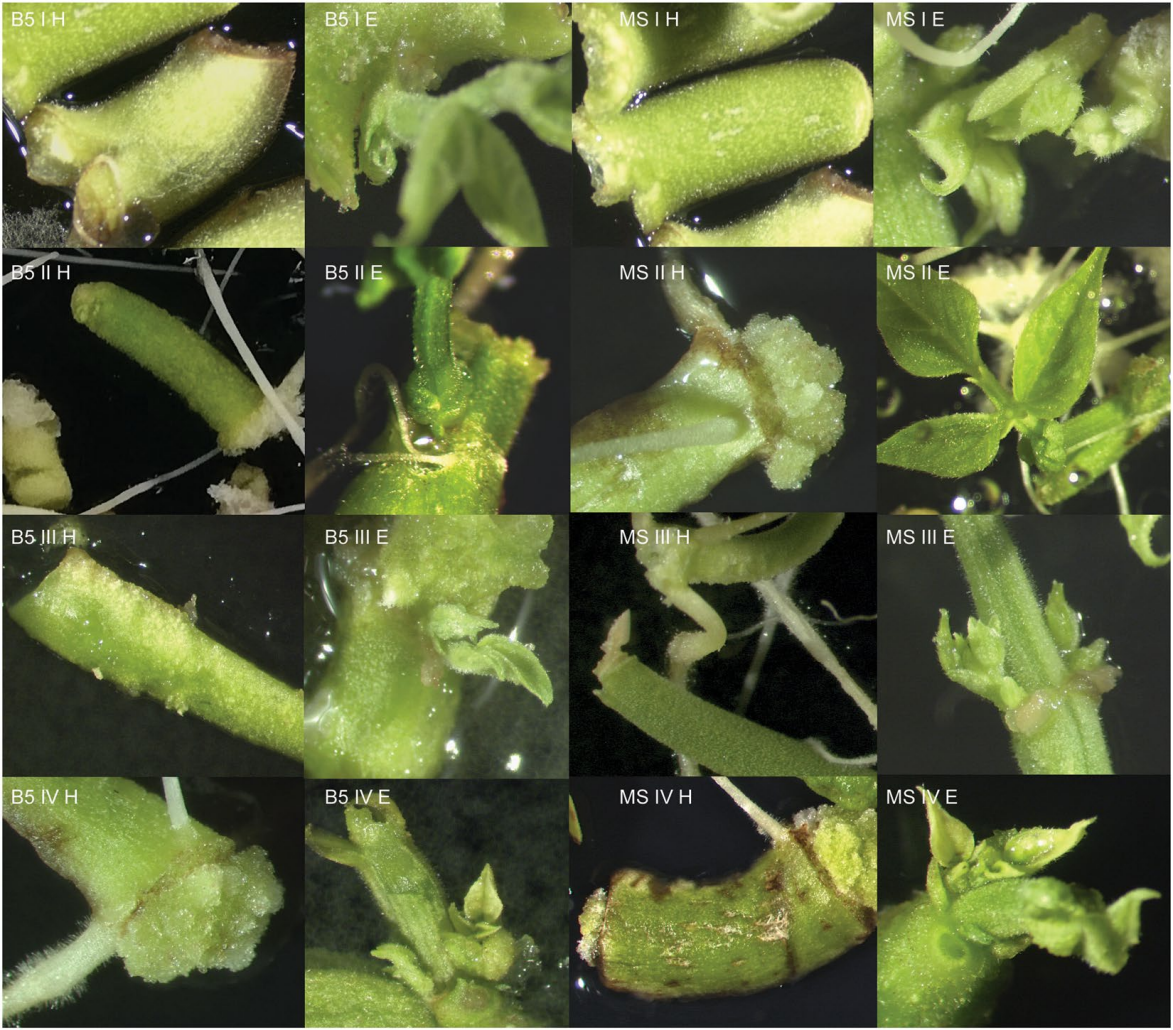
*In vitro* response of the hypocotyls and epicotyls of the Ibiza cultivar on different media. B5 I H – Gamborg Medium, variant I, hypocotyl; B5 II H – Gamborg Medium, variant II, hypocotyl; B5 III H – Gamborg Medium, variant III, hypocotyl; B5 IV H – Gamborg Medium, variant IV, hypocotyl; B5 I E – Gamborg Medium, variant I, epicotyl; B5 II E – Gamborg Medium, variant II, epicotyl; B5 III E – Gamborg Medium, variant III, epicotyl; B5 IV E – Gamborg Medium, variant IV, epicotyl; MS I H – Murashige-Skoog Medium, variant I, hypocotyl; MS II H – Murashige-Skoog Medium, variant II, hypocotyl; MS III H – Murashige-Skoog Medium, variant III, hypocotyl; MS IV H – Murashige-Skoog Medium, variant IV, hypocotyl; MS I E – Medium, variant I, epicotyl; MS II E – Murashige-Skoog Medium, variant II, epicotyl; MS III E – Murashige-Skoog Medium, variant III, epicotyl; MS IV E – Murashige-Skoog Medium, variant IV, epicotyl.

**Figure 5:**
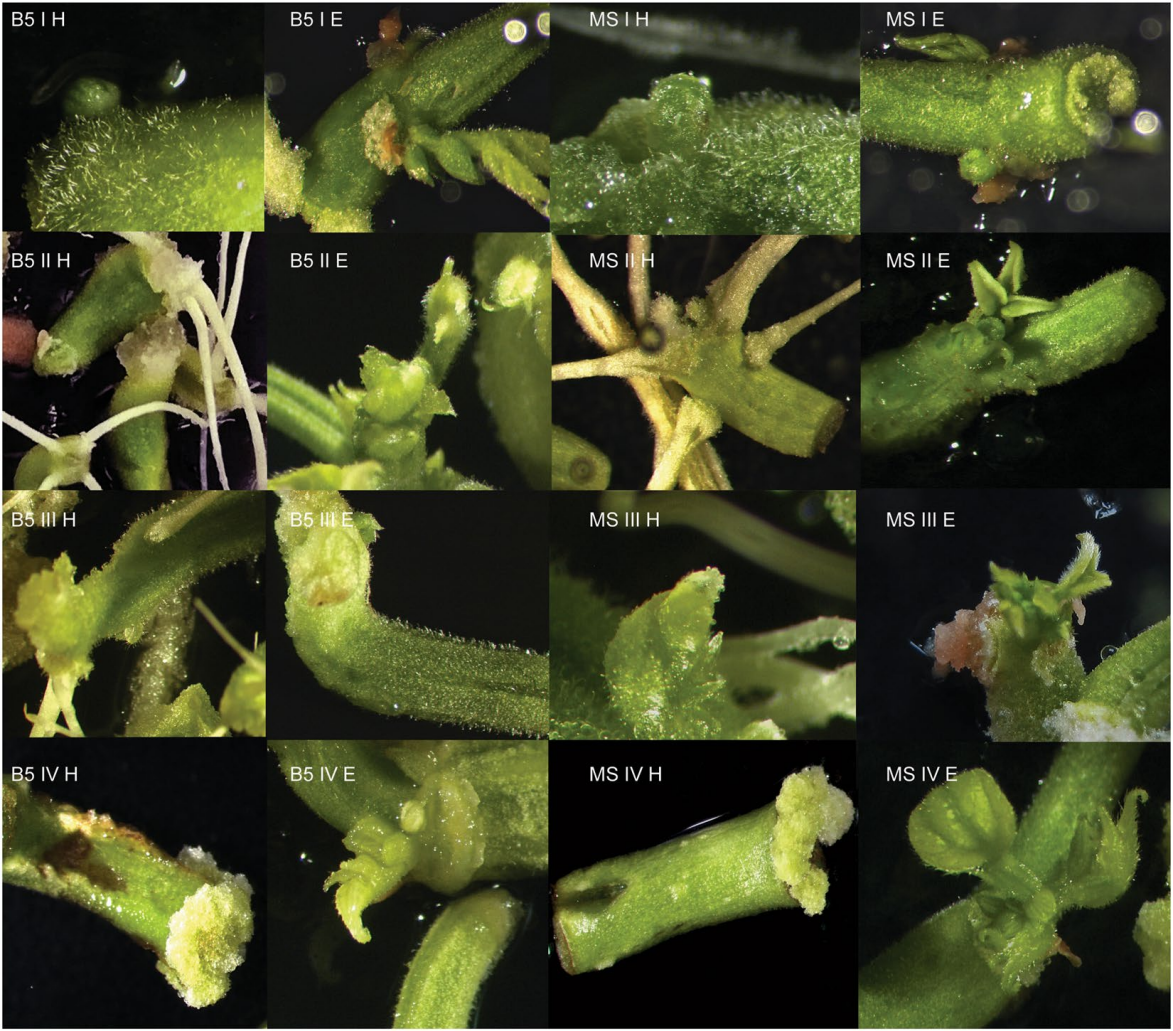
*In vitro* response of the hypocotyls and epicotyls of the Plus cultivar on different media. B5 I H – Gamborg Medium, variant I, hypocotyl; B5 II H – Gamborg Medium, variant II, hypocotyl; B5 III H – Gamborg Medium, variant III, hypocotyl; B5 IV H – Gamborg Medium, variant IV, hypocotyl; B5 I E – Gamborg Medium, variant I, epicotyl; B5 II E – Gamborg Medium, variant II, epicotyl; B5 III E – Gamborg Medium, variant III, epicotyl; B5 IV E – Gamborg Medium, variant IV, epicotyl; MS I H – Murashige-Skoog Medium, variant I, hypocotyl; MS II H – Murashige-Skoog Medium, variant II, hypocotyl; MS III H – Murashige-Skoog Medium, variant III, hypocotyl; MS IV H – Murashige-Skoog Medium, variant IV, hypocotyl; MS I E – Murashige-Skoog Medium, variant I, epicotyl; MS II E – Murashige-Skoog Medium, variant II, epicotyl; MS III E – Murashige-Skoog Medium, variant III, epicotyl; MS IV E – Murashige-Skoog Medium, variant IV, epicotyl.

**Figure 6:**
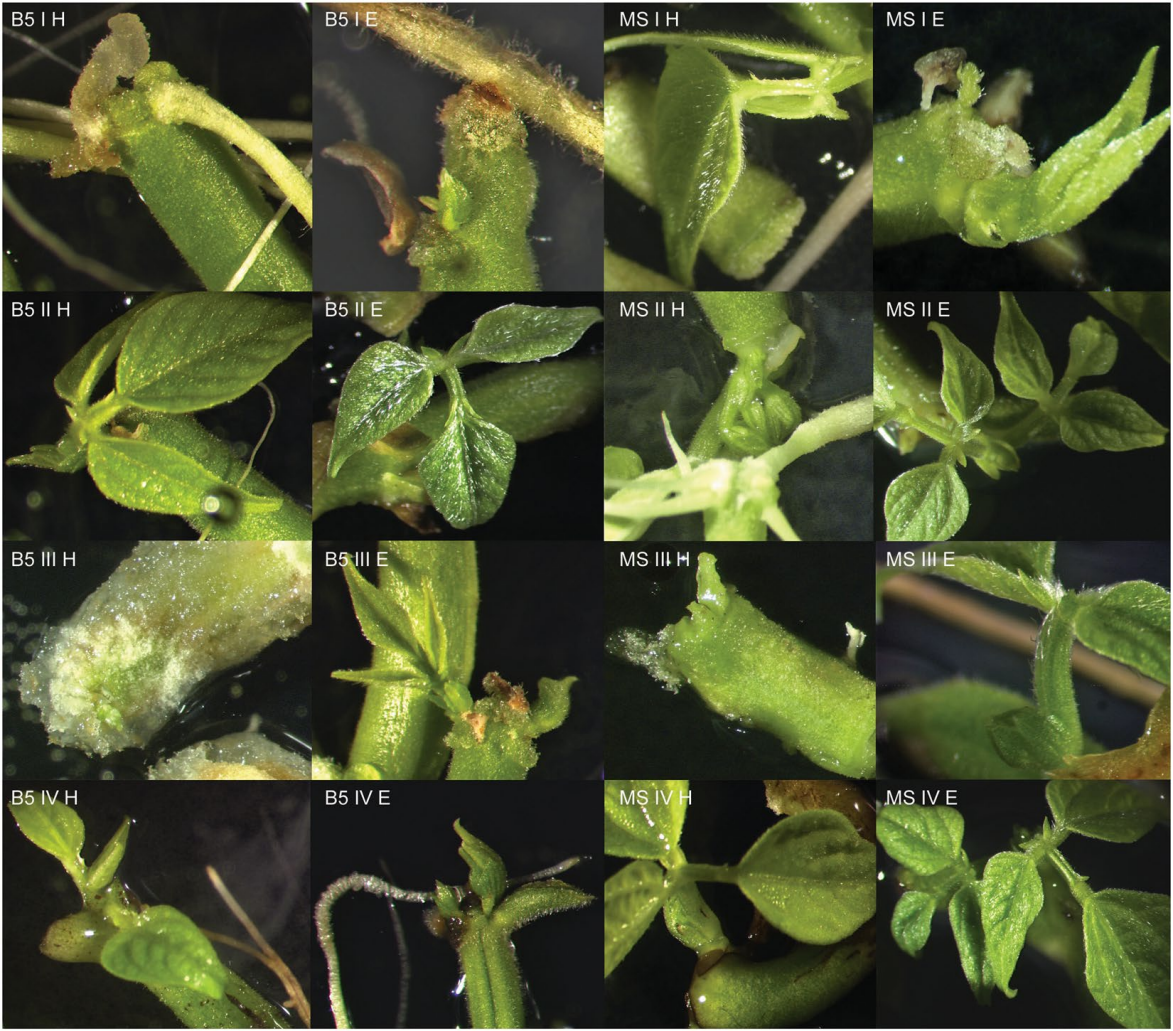
*In vitro* response of the hypocotyls and epicotyls of the Czerwona cultivar on different media. B5 I H – Gamborg Medium, variant I, hypocotyl; B5 II H – Gamborg Medium, variant II, hypocotyl; B5 III H – Gamborg Medium, variant III, hypocotyl; B5 IV H – Gamborg Medium, variant IV, hypocotyl; B5 I E – Gamborg Medium, variant I, epicotyl; B5 II E – Gamborg Medium, variant II, epicotyl; B5 III E – Gamborg Medium, variant III, epicotyl; B5 IV E – Gamborg Medium, variant IV, epicotyl; MS I H – Murashige-Skoog Medium, variant I, hypocotyl; MS II H – Murashige-Skoog Medium, variant II, hypocotyl; MS III H – Murashige-Skoog Medium, variant III, hypocotyl; MS IV H – Murashige-Skoog Medium, variant IV, hypocotyl; MS I E – Murashige-Skoog Medium, variant I, epicotyl; MS II E – Murashige-Skoog Medium, variant II, epicotyl; MS III E – Murashige-Skoog Medium, variant III, epicotyl; MS IV E – Murashige-Skoog Medium, variant IV, epicotyl.

**Figure 7:**
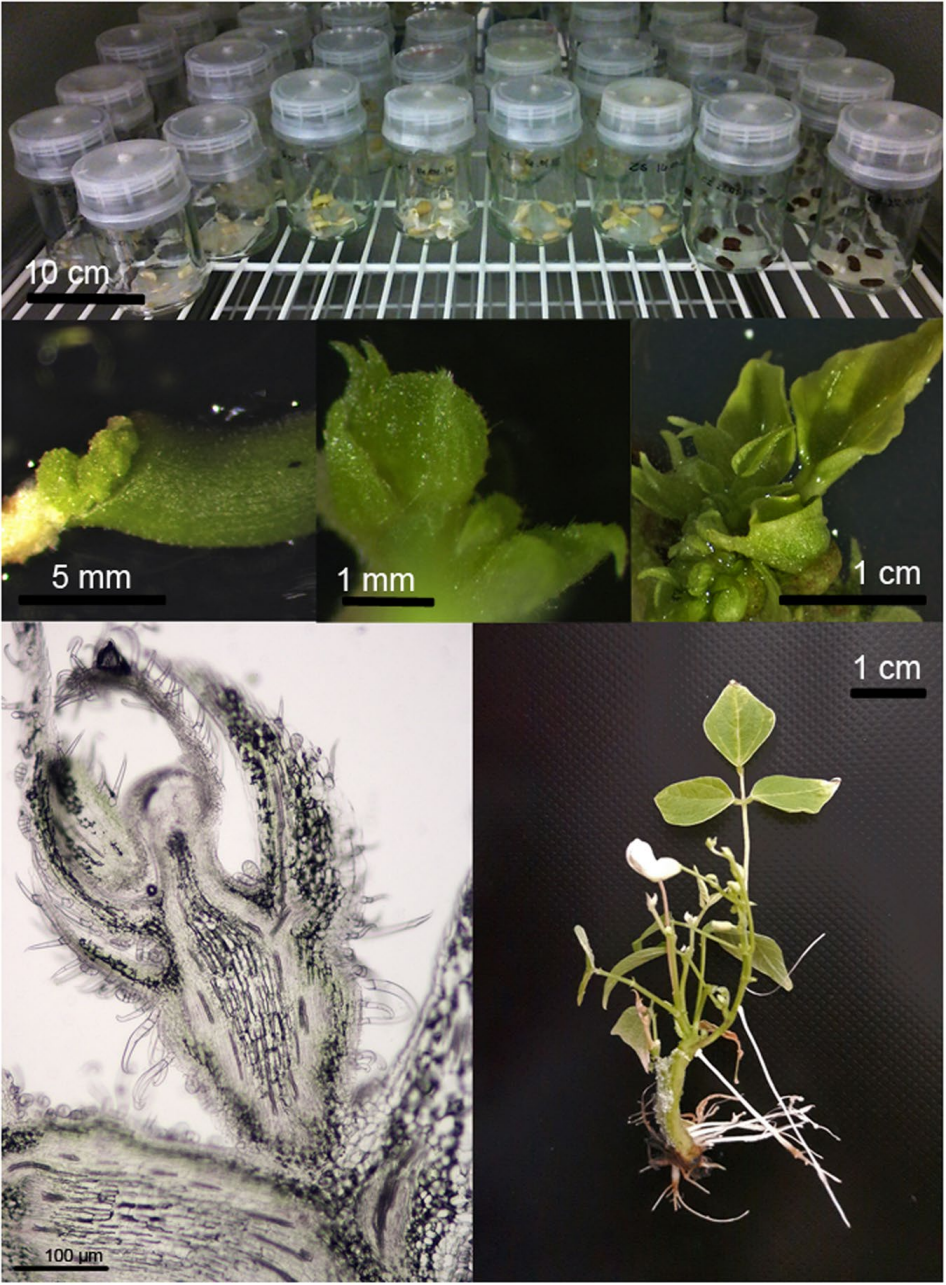
Stages of regeneration of the common bean cultivar (Czerwona): (**A**) seed germination; (**B**) non-morphogenic callus; (**C**) bud induction; (**D**) cluster of shoots on epicotyl explant; (**E**) microscopy analysis: the shoot apical meristem formed on the surface of epicotyl. The apical dome is surrounded by leaf primordia and young leaves encircling them. The vascular strand with noticeable procambium fills the central part of the formed new stem and furcates to young leaves. The parenchyma cells beneath epidermis contain chloroplasts. The epidermis is fully developed with two types of trichomes. Thin stright trichomes predominate but clubbed multicellular glandular trichomes are also quite numerous; (**F**) regenerated and rooted *Phaseolus vulgaris* plant.

The common bean cultivars evaluated in this study differed in the average number of buds induced. The efficiency of regeneration was calculated as the number of explants with buds induced per total of explants tested. The results are presented in the Tables and Figures below (Tables 2–7, Figs 1–6). In this research regeneration efficiency of 0–70% was achieved, depending on the cultivar. The first symptom of the regeneration process appeared after 8–10 days, and the regeneration potential was strongly connected with the explant type, pointing to the undisputed superiority of epicotyls. The highest shoot bud formation, regardless of the basal medium type, was obtained with Czerwona epicotyls (70.0% ± 9.26). The stages of regeneration of the Czerwona cultivar are presented in Fig. 7. The maximum regeneration efficiency achieved for hypocotyls was 31.67% (Laponia). The regeneration responses varied depending on both the type of basal medium tested and the composition of the medium. Among the six cultivars, three showed the highest *in vitro* response on the Gamborg medium and three on the Murashige-Skoog medium (considering their percentage absolute value). Direct organogenesis appeared to be the dominant regeneration pathway with the callus appearing only at the ends of explants; the indirect pathway was observed sporadically.

**Table 2:** Goldpantera cultivar – the results are representative of three replicates and are expressed as means ± SE.

T1	Gamborg Medium		Murashige & Skoog Medium	
	Hypocotyls	Epicotyls	Hypocotyls	Epicotyls
I	6.67% ± 1.95 fg	19.44% ± 2.00 cd	0.0% ± 0	22.7% ± 1.47 c
II	10.0% ± 1.47 ef	23.33% ± 2.31 c	11.11% ± 3.06 ef	20.0% ± 3.47 cd
III	13.89% ± 2.55 de	**58**.**89%** ± **1**.**47 a**	0.0% ± 0	35.0% ± 2.10 b
IV	2.78% ± 2.00 h	16.11% ± 2.63 de	1.11% ± 0.64 h	13.89% ± 0.85 de

The means with the same letter do not differ significantly according the post-hoc Tukey’s test (p ≤ 0.05).

**Table 3:** Złota Saxa cultivar – the results are representative of three replicates and are expressed as means ± SE.

T2	Gamborg Medium		Murashige & Skoog Medium	
	Hypocotyls	Epicotyls	Hypocotyls	Epicotyls
I	14.44% ± 4.17 cd	**46**.**11%** ± **4**.**10 a**	6.67% ± 1.40 de	**42**.**78%** ± **2**.**25 a**
II	8.89% ± 1.15 de	34.44% ± 3.64 b	4.44% ± 1.79 e	25.0% ± 7.33 bc
III	12.22% ± 2.55 cd	31.67% ± 1.95 b	0.19% ± 0.19 f	**33**.**33%** ± **4**.**34 ab**
IV	22.78% ± 3.06 c	30.0% ± 1.92 b	12.22% ±± 1.28 cd	**31**.**67%** ± **3**.**06 ab**

The means with the same letter do not differ significantly according to the post-hoc Tukey’s test (p ≤ 0.05).

**Table 4:** Laponia cultivar – the results are representative of three replicates and are expressed as means ± SE.

T3	Gamborg Medium		Murashige & Skoog Medium	
	Hypocotyls	Epicotyls	Hypocotyls	Epicotyls
I	0.0% ± 0	0.0% ± 0	0.0% ± 0	13.89% ± 2.79 c
II	31.67% ± 3.78 b	**47**.**22%** ± **3**.**06 a**	12.78% ± 8.63 cd	**44**.**44%** ± **4**.**79 ab**
III	17.78% ± 2.74 c	13.89% ± 4.63 c	0.0% ± 0	**42**.**78%** ± **3**.**96 ab**
IV	15.56% ± 1.47 c	2.78% ± 1.47 d	10.0% ± 2.42 c	**50**.**56%** ± **14**.**24 ab**

The means with the same letter do not differ significantly according to the post-hoc Tukey’s test (p ≤ 0.05).

**Table 5:** Ibiza cultivar – the results are representative of three replicates and are expressed as means ± SE.

T4	Gamborg Medium		Murashige & Skoog Medium	
	Hypocotyls	Epicotyls	Hypocotyls	Epicotyls
I	0.0% ± 0	40.0% ± 1.79 b	0.0% ± 0	42.78% ± 2.42 b
II	1.11% ± 0.56 e	35.0% ± 2.56 bc	0.0% ± 0	**58**.**89%** ± **8**.**73 a**
III	3.33% ± 1.93 e	19.44% ± 2.91 d	0.74% ± 0.74 e	25.0% ± 10.60 cd
IV	0.0% ± 0	33.89% ± 0.64 c	0.0% ± 0	**50**.**0%** ± **1**.**95 a**

The means with the same letter do not differ significantly according to the post-hoc Tukey’s test (p ≤ 0.05).

**Table 6:** Plus cultivar – the results are representative of three replicates and are expressed as means ± SE.

T5	Gamborg Medium		Murashige & Skoog Medium	
	Hypocotyls	Epicotyls	Hypocotyls	Epicotyls
I	2.22% ± 0.55 f	30% ± 2.1 c	1.11% ± 0.64 f	23.33% ± 1.79 c
II	1.11% ± 0.55 f	**43**.**89%** ± **4**.**06 ab**	0.0% ± 0	33.89% ± 3.38 bc
III	4.44% ± 2.50 ef	10.0% ± 1.16 de	4.44% ± 1.79 ef	12.22% ± 1.63 d
IV	0.0% ± 0	**55**.**56%** ± **2**.**42 a**	0.0% ± 0	**43**.**89%** ± **3**.**39 ab**

The means with the same letter do not differ significantly according to the post-hoc Tukey’s test (p ≤ 0.05).

**Table 7:** Czerwona cultivar – the results are representative of three replicates and are expressed as means ± SE.

T6	Gamborg Medium		Murashige & Skoog Medium	
	Hypocotyls	Epicotyls	Hypocotyls	Epicotyls
I	0.0% ± 0	5.0% ± 1.28 f	22.78% ± 5.74 cd	18.89% ± 2.11 d
II	30.0% ± 3.35 c	42.22% ± 2.80 b	16.11% ± 4.01 de	47.22% ± 2.37 b
III	6.67% ± 0.64 f	26.67% ± 3.16 c	6.67% ± 1.47 f	30.0% ± 1.16 c
IV	12.22% ± 1.95 e	22.78% ± 9.54 cde	11.67% ± 1.16 e	**70**.**0%** ± **9**.**26 a**

The means with the same letter do not differ significantly according to the post-hoc Tukey’s test (p ≤ 0.05).

The highest rooting efficiency was achieved for Zlota Saxa (66.0% ± 3.83, the cultivar that showed the lowest maximal regeneration potential). Except for Czerwona, the type of basal medium seems to have no influence on the rooting process. The rooting efficiency is presented in Table 8. Fully developed *Phaseolus vulgaris* plants presented a similar phenotype to the control plants obtained *via* standard germination. A brief analysis of the probable regeneration preferences of the epicotyl explants of particular cultivars is presented in Table 9.

**Table 8:** The results are representative of three replicates for *Phaseolus vulgaris* cultivars rooted on B5 and MS media and are expressed as means ± SE.

*cultivar*	Gamborg Medium	Murashige & Skoog Medium
Goldpantera	40.0 ± 2.11 cd	44.33 ± 3.22 bc
Złota Saxa	**63**.**67** ± **2**.**64 a**	**66**.**0** ± **3**.**83 a**
Laponia	46.0 ± 3.26 bc	42.0 ± 3.51 bcd
Ibiza	25.0 ± 5.20 e	22.0 ± 4.09 e
Plus	31.0 ± 3.65 de	34.0 ± 3.51 de
Czerwona	51.67 ± 2.05 b	42.0 ± 3.09 bcd

The means with the same letter do not differ significantly according to the post-hoc Tukey’s test (p ≤ 0.05).

**Table 9:** The analysis of the probable regeneration preferences of the epicotyls of the particular cultivars.

cultivar	regeneration efficiency (RE) to basal medium and to additional phytoregulators
Goldpantera	no preferences regarding the basal medium for variants I, II and IV → no statistically significant differences between B5 I and MS I, B5 II and MS II, B5 IV and MS IV; two the highest *RE* results were clearly stimulated by the supplementation with the highest concentration of BAP (variant III), however the maximum *RE* was achieved on B5 medium → *the role of basal medium is secondarily stressed by a particular compound in an optimal concentration* (? here BAP?)
Złota Saxa	no preferences regarding phytoregulators for variants II, III and IV → no statistically significant differences observed both, in corresponding pairs of variants (B5 II and MS II, B5 III and MS III, B5 IV and MS IV) and among all these variants (e.g B5II and MS IV); similarly, no statistically significant differences observed between variants B5 I and MS I, however the RE values for pure B5 and MS were significantly higher than all the others → pure basal media stimulated the highest RE; the addition of any of the tested phytoregulators seemed to lower it (*interfering medium balance*?)
Laponia	the highest *ER* results were observed mostly when MS medium was employed (among B5 variants tested only B5 II brought one out of four highest values of the *RE* giving rise to certain inconsistency that is difficult to explain) → statistically significant differences were recorded between three (out of four) pairs of B5 and MS variants (B5 I and MS I, B5 III and MS III, B5 IV and MS IV); on the other hand when only MS medium influence was analysed, the addition of phytoregulators seemed significant for better results, however their character did not have to be so defined → the RE here was stimulated by the medium type itself and this stimulation may be strengthen by additional growth factors (*crucial role of the basal medium in combination with a growth regulator of less dedicated character*?)
Ibiza	no preferences regarding basal medium for variants I and III → no statistically significant differences observed, in the other cases differences in variants were significant, clearly favouring MS medium; the highest *RE* results were achieved on MS II and MS IV suggesting beneficial effect of IAA or AgNO_3_ and low concentration of BAP → the regeneration potential seemed to be equally stimulated by combination of auxin and cytokinin as by combination of cytokinin and silver nitrate → *crucial role of basal medium in combination with a growth regulator of less dedicated character* (?)
Plus	no preferences regarding basal medium when variants I and III tested → no statistically significant differences between B5 I and MS I, B5 III and MS III → pure basal media or high concentration of BAP did not stimulate increase in *RE* (in fact the addiction of the latter seems to lower *RE*); the comparison of variants II indicates the role of IAA and low concentration of BAP, however better results were achieved with B5 medium; similarly, the comparison of variants IV indicated the role of AgNO_3_ and BAP (low concentration), and again the better result was achieved with B5, stressing the role of the medium; on the other hand the comparison of B5 II and IV stressed the influence of silver nitrate that brought the highest *REs* → B5 + AgNO_3_ + BAP seems to be the right (dedicated?) composition → (*crucial role of the combination of the basal medium and the particular compound*? here B5/AgNO_3_?)
Czerwona	higher *RE* on MS while responding to the pure basal medium (variants I); the supplementation with hormones caused the increase in *RE* in comparison to variants I, however the addition of IAA and the low concentration of BAP (variants II) or just high concentration of BAP (variants III) seemed to level the initial role of the basal media (no statistically significant differences between B5 II, B5 III, MS II and MS III); MS + AgNo3 + BAP seems to be the right (dedicated?) composition (*crucial role of the combination of the basal medium and the particular compound*? here MS/AgNo3?)

## Discussion

The need for an approach that brings efficient shoot formulation and regeneration of the whole plant is a crucial requirement for the genetic modification of plants. The goal of this study was to develop a reliable and efficient regeneration protocol for common bean plants. Previously published procedures were used as an initial tool to generate it. It was already noticeable at the level of preliminary research that not all of these protocols were universal and directly adoptable. This was the case with Kwapata’s *et al*. protocol^8^. Of course, this could be due to the type of explants that were chosen for the investigation. Ahmed *et al*.^16^ used intact seedlings and cotyledonary nodes, Delgado-Sanchez *et al*.^2^ and Kwapata *et al*.^8^ used embryonic axes. Both hypocotyls and epicotyls chosen by us represent a later developmental stage than embryo axes. It may therefore result in differences in the *in vitro* response. As a result, significant differences (p ≤ 0.05) were observed in the rates of organogenic bud formation among the two types of explants of six cultivars grown on different variations of the Gamborg or Murashige and Skoog medium. Based on the values obtained, several observations could be expressed.

First of all, the chosen parts of seedlings displayed varied regeneration potential. The area of the epicotyls (especially the junction of the cotyledonary node and the epicotyl; and the neighbouring tissues of the meristems of the apical and axillary buds) showed undisputed superiority over the hypocotyls. The capacity of epicotyls to become a bud source was also demonstrated by Collado *et al*.^17^, who used them as transformation material. Hypocotyls do not seem to be the recommended explants for efficient propagation of the common bean. Among the total number of 48 approaches (six cultivars, four media variants, two basal media) to bud induction from hypocotyl tissues, there were 12 events with no response observed and 16 events with an assessment of regeneration capacity below 10%. Their maximum induction efficiency reached a level of 31.67% (Laponia). It seems that in the case of the common bean, seedling epicotyls and hypocotyls in particular follow their natural “differentiation guidebook” rather than make use of their totipotency. A predisposition to root formation that is characteristic of seed legumes^18^ was also revealed in our research. It was exceptionally well demonstrated when hypocotyl explants were investigated. Again, this seems more natural since hypocotyls realize their genetic program giving rise to the lower stem and, along with radicles, to the roots.

Secondly, in the case of epicotyls we could not agree with the conclusions by Quintero-Jiménez *et al*.^6^ that the Murashige and Skoog medium was typically less effective than the Gamborg one (irrespective of the concentration of growth phytoregulators). Quintero-Jiménez’s team reported the high efficiency of organogenic shoot formation (98–100%) induced by B5 and lower efficiency (15–73%) induced by MS^6^. It was supposed to stress the importance of the differences in the concentration of macroelements between MS and B5. The results presented here (concerning more cultivars) seem rather consistent with those from a previous publication by the same scientific group (Delgado-Sánchez *et al*. in)^2^, where 18% to 90% bud formation on MS was obtained. In fact, inconsistent organogenic bud/shoot formation could be observed on the MS medium in all the experiments - in our study, in the study by Delgado-Sánchez *et al*.^2^ and in the study by Quintero-Jiménez *et al*.^6^. However, in contrast to the latter, we did not observe the postulated consistency when B5 medium was used (0–58.89%). Even the statistical analysis of the response rates of epicotyls (explant of a higher regeneration potential) obtained on the basal media without phytohormones (variant I) did not clearly indicate their strict correlation with the macroelement differences between MS and B5 (in most cases the same statistical label/letter could be seen). Hence, the concentration of macroelements is indeed crucial for the *in vitro* response^6^, although this response is directed by the genotype. Unfortunately, from the point of view of scientific versatility, all this seems to strongly support Kwapata *et al*.’s^8^ conclusion that common bean regeneration is cultivar-specific or at least specific to the cluster of related cultivars. On the other hand, when investigating three cultivars (Laponia, Plus, Casablanca) in 2014 we did agree with the observations made by Quintero-Jiménez *et al*.^1,6^. Interestingly, at that time we investigated the hypocotyl response. The new results seem to be quite consistent with the previous ones. Four out of six cultivars showed the highest hypocotyl response on the B5 medium; for one cultivar no statistically significant difference was noted. Moreover, nine out of the twelve aforementioned events of negative (zero) response took place when MS medium was used. If a certain correlation between hypocotyls and the basal medium shows here, perhaps an explant-specific composition of the medium for *Phaseolus vulgaris* may be considered as well.

Thirdly, the highest bud formation was obtained for Czerwona on MS. Regarding the highest values of the *in vitro* response (irrespective of the statistical analysis and medium type), the regeneration potential could be presented as follows: Czerwona (70.00%) > Goldpantera (58.89%) and Ibiza (58.89%) > Plus (55.56%) > Laponia (50.56%) > Złota Saxa (46.11%). It could be concluded that except for “the best” and “the worst” cultivar, perhaps, no spectacular differences were noted. However, they were obtained on different media. For example, Goldpantera (58.89%) achieved its highest regeneration efficiency on variant III of B5, while with Ibiza (58.89%) it was on variant II of MS. These cultivars responded differently not only to the basal medium type, but to the presence of various additional components as well. Such variations are clearly visible when analysing the outcomes. Some cultivars seem to require a dedicated composition, causing the level of the *in vitro* response to be considerably higher than that obtained on other medium variants (e.g. for Czerwona such a dedicated medium would seem to be variant IV of B5; on the other media, the efficiency is lower). This analysis of the probable regeneration preferences of the epicotyl explants of particular cultivars was briefly described in Table 9 in the Results section.

In spite of the complexity of the regeneration capacity of the cultivars of the common bean, it could be noted that for five out of all the cultivars tested, variant IV, regardless of the basal medium type, proved most effective (taking into account statistical significance). The beneficial effect of the synergistic impact of BAP and AgNO_3_ was described by Kwapata *et al*.^8^. In 2000 Cruze de Carvalho *et al*. reported 100% of developed shoots from transverse thin cell layers (tTCLs) from 2-week-old seedlings when silver nitrate was applied with BAP^19^. When used alone, the results were 63.8% of developed shoots for cytokinin and 51.3% of shoot formation for AgNO_3_. Since the combination of BAP and IAA (our variant II) promoted the highest frequencies rather seldom, the primacy of variant IV could be due to the presence of an ethylene action inhibitor – silver nitrate. Ethylene synthesis is considered to be responsible for poor common bean regeneration. It was reported to negatively influence the regeneration of shoots, callus growth and somatic embryogenesis. Plants decrease it by using weak and strong ethylene antagonists (such as CO_2_ and silver compounds respectively). Given its lack of phytotoxicity at working concentrations and water solubility, the application of silver nitrate to modulate ethylene action in tissue cultures seems justified^20^. For instance, Sgamma *et al*.^21^ showed that AgNO_3_ enhanced the regeneration potential of young leaves of *Prunus avium*.

The positive response on medium IV may also result from the presence of activated charcoal. To overcome the problem of synthesing phenolic compound Kwapata *et al*.^8^ tested the importance of silver nitrate and activated charcoal separately and proved their beneficial effect with the former being more efficient. It is possible that their simultaneous supplementation in our study could have a positive synergistic influence on bud formation in common bean (however, we did not test their separate effects on our cultivars). In general, it seems likely that the application of activated charcoal to all the media variants assured the proper quality of explants. The characteristic browning of plant tissues was hardly observed, even when cytokinin (BAP) was used (variant III), which is known to have the potential for positive correlation with the synthesis of phenolic compounds^3,22^.

The investigation of the rooting capacity was conducted at a preliminary level, since the viability of plants was quite poor. The rooting efficiency determined in epicotyl-derived shoots ranged from 25% to 66% (evaluated after three weeks). The type of basal medium seemed to have no influence on the rooting process of the cultivars that were tested, showing no statistically significant differences. The only exception was Czerwona, which achieved a noticeably higher value on the B5 rooting medium (while its highest bud regeneration was observed on MS). It should be pointed here that the influence of specific variants of the basal media on the rooting efficiency was not evaluated. We did not follow the further growth and rooting of cultivars regarding particular medium variants. Since the investigation of such a correlation would be interesting, it will be the subject of our next study.

By way of conclusion, the tissue culture of *Phaseolus vulgaris* has repeatedly been found to be difficult, hampering the application of genetic engineering. Here, we recommend a repeatable technique for organogenic bud induction from epicotyls after having investigated six cultivars of the common bean and four variants of two basal media (Murashige-Skoog and Gamborg). The most effective regeneration was achieved for the MS or B5 media amended with AgNO_3_ and BAP. However, it is still too soon to consider it universal. Unfortunately, it seems that we confirmed a well-known observation that common bean regeneration is specific, at the least for the cluster of related cultivars. Regarding the role of the basal medium type, it seems to be genotype-dependent as well. For some cultivars the type of basal medium becomes important only after supplementation with a particular compound. Additionally, the positive synergistic influence of activated charcoal and silver nitrate on bud formation was demonstrated.

## Material and Methods

### Material

#### Plant material

Six cultivars of common bean were employed: Czerwona, Laponia, Złota Saxa, Ibiza, Goldpantera and Plus (*Plantico*, Poland). Seeds were surface-sterilized in 70% ethanol for 1 min and in a 10% sodium hypochloride solution for 10 min, followed by five rinses with sterile-distilled water. The seeds were placed in a sterile jar and maintained in the dark for 5 days, then they were transferred to light, where they remained for the next 2 days (25 °C with a 16-h photoperiod).

#### Media

The induction–multiplication media are presented in Table 1. All medium variants containing vitamins (MS I–IV, *Duchefa*, and B5 I–IV, *Duchefa*) were supplemented with 500 mg/L cefotaxime and solidified with 0.8% agar. The rooting medium consisted only of B5^23^ or MS^24^ basal medium supplemented as above. Since we found the supplementation with absorbent quite beneficial for the common bean regeneration, the activated charcoal became a basal part of the medium *formula* both at the stage of shoot multiplication and rooting. All media were autoclaved at 121 °C for 20 min and poured into Petri dishes.

The rooting medium had the same composition as the basal induction–multiplication medium (variant I), excluding growth regulators.

### Methods

Five seeds were placed in every jar for germination to obtain minimum 90 seedlings for a particular cultivar. Next, the cylinders of both the hypocotyl and epicotyl area were cut along their length to obtain two fragments of the hypocotyl and two fragments of the epicotyl per seedling. The common bean explants were split up among four variants of the induction–multiplication medium. The first evidence of the regeneration process appeared after 8–10 days. All explants were transferred to a new medium every 14 days and cultured in a 16-h photoperiod at 23 °C. The rooting was induced as soon as the shoots reached 1–1.5 cm in length.

Experimental treatments consisted of three replicates of 180 explants of a given explant type, resulting in a total of 540 hypocotyls and 540 epicotyls per medium variant per cultivar.

#### Microscopic analysis

Samples were sectioned and observed on the day of material collection. The material was embedded in 5% (w/v) agarose. A VT1200S vibrating blade microtome (Leica Microsystems, Germany) was used to produce 25 µm thick sections. The bright-field microscopic imaging of sections was performed using an Eclipse 50i upright microscope equipped with an Fi3 camera and NIS D imaging software (Nikon Instruments, Netherlands). These analyses were performed in the Laboratory of Microscopic Imaging and Specialized Biological Techniques at the Faculty of Biology and Environmental Protection at the University of Lodz.

#### Statistical analysis

All results are presented as the mean ± standard error (SE). The results were analyzed using one way ANOVA, followed by the post-hoc Tukey’s test for multiple comparisons. The level of significance was set at 5%. STATISTICA 13.1 (STATSoft, Poland) software was used for calculations.

## Data Availability

No datasets were generated or analysed during the current study.
